# Measuring geometric accuracy in magnetic resonance imaging with 3D-printed phantom and nonrigid image registration

**DOI:** 10.1007/s10334-019-00788-6

**Published:** 2019-10-23

**Authors:** Katri Nousiainen, Teemu Mäkelä

**Affiliations:** 1 HUS Medical Imaging Center, Radiology, Helsinki University Hospital and University of Helsinki, Helsinki, Finland; 2 Department of Physics, University of Helsinki, Helsinki, Finland

**Keywords:** Magnetic resonance imaging, Artifacts, Quality control, Healthcare quality assurance

## Abstract

**Objective:**

We aimed to develop a vendor-neutral and interaction-free quality assurance protocol for measuring geometric accuracy of head and brain magnetic resonance (MR) images. We investigated the usability of nonrigid image registration in the analysis and looked for the optimal registration parameters.

**Materials and methods:**

We constructed a 3D-printed phantom and imaged it with 12 MR scanners using clinical sequences. We registered a geometric-ground-truth computed tomography (CT) acquisition to the MR images using an open-source nonrigid-registration-toolbox with varying parameters. We applied the transforms to a set of control points in the CT image and compared their locations to the corresponding visually verified reference points in the MR images.

**Results:**

With optimized registration parameters, the mean difference (and standard deviation) of control point locations when compared to the reference method was (0.17 ± 0.02) mm for the 12 studied scanners. The maximum displacements varied from 0.50 to 1.35 mm or 0.89 to 2.30 mm, with vendors’ distortion correction on or off, respectively.

**Discussion:**

Using nonrigid CT–MR registration can provide a robust and relatively test-object-agnostic method for estimating the intra- and inter-scanner variations of the geometric distortions.

## Introduction

Magnetic resonance imaging (MRI) has a superior soft tissue contrast in comparison to computed tomography (CT). Consequently, MRI is nowadays used alone or together with CT in the treatment planning and guidance of many medical operations, such as stereotactic radiotherapy, stereotactic surgery, and stereoelectroencephalography implantation. These operations require excellent geometric accuracy; the required precision of radiosurgery is of sub-millimeter scale [1]. However, magnetic resonance (MR) images suffers from geometric distortions that can degrade this accuracy. The system-related geometric distortions in MRI arise from inherent magnetic field inhomogeneities and gradient coil nonlinearities [2]. The nonlinearity of the gradient coils has become more relevant as modern scanners have strong gradients with short rise times. The inhomogeneity of the main magnetic field is emphasized in scanners with short bores and high field strengths. In addition, the chemical shift and the susceptibility difference can cause patient-induced distortions; this paper only assesses the system-related factors of the geometric distortions.

Studying the geometric accuracy of MRI has been a subject of great interest in recent decades, especially from the aspect of quality assurance (QA) [3–6], radiotherapy treatment planning (RTP) [7–16], and stereotactic operations [17–19]. Most MRI QA protocols include at least a limited investigation of the geometric accuracy, often reporting deviations from known phantom structure lengths. An example of such a protocol is the MRI accreditation program of American College of Radiology (ACR) that is performed with their cylindrical multipurpose phantom [20]. A comprehensive description of the distortions can be provided with a vector field, with the magnitude and the direction of the geometric displacements inside a physical volume-of-interest. Such measured displacement fields can be applied for QA, as well as to “unwarp” patient images [9].

Studies about the system-related distortions are often performed with phantoms designed and built for the purpose, although commercial products are also available. Generally, the phantoms have a regular structure; either a hyposignal grid in a signal-producing background medium [3, 6–10], or a pattern of signal-producing markers in a hyposignal medium [4, 5, 11–13, 17]. Typically, the signal-producing material is either water–salt solution [3, 5, 7, 19], paraffin oil [6, 8–10, 17] or fat-soluble vitamin capsules [11, 12]. For example, Wang et al. [3] built a phantom from grid sheets, where the interfaces of the water-solution medium and the crosses in the grid sheet served as control points (CP). Jafar et al. [6] 3D printed a grid and filled their phantom with off-the-shelf baby oil.

A majority of the studies above are based on locating CPs (i.e. the grid vertices or other markers) in the MR images and comparing the locations to a ground truth. The ground truth is often a CT acquisition of the same phantom [6, 8, 19]. The CP localization is achieved with image-processing tools, such as edge detection, convolution, binarization, morphological operations, and center-of-mass calculation. After locating and pairing the CPs in the MR image and the ground truth, a displacement field, and sometimes distortion correction, is produced through interpolation or spherical harmonics [e.g. 4, 5].

Many of the existing phantoms, especially those developed for RTP purposes, are large field-of-view phantoms [4, 10–12] and, consequently, scanned with table-integrated spine coils. Not many phantoms are designed for head coils: the phantom of Vermandel and Betrouni [17] can be modified to fit into both head and body coils; Zhang et al. [18] and Pappas et al. [19] used Leksell Stereotactic frame (Elekta Instruments AB, Stockholm, Sweden) that stabilizes their phantoms but inflicts distortions, too.

Whereas most previous studies have used interpolation methods to determine the displacement fields, Baldwin et al. [8], Sun et al. [10] and Walker et al. [11] implemented nonrigid image registration. In addition, Gustafsson et al. [14] and Adjeiwaah et al. [15] used a commercially available phantom and software GRADE (Spectronic Medical AB, Helsingborg, Sweden) that utilizes nonrigid registration. However, to our knowledge, only Walker et al. applied the nonrigid registration to the plain image data and not to the already localized CP pairs of the MR and ground truth images.

This study evaluates, if a nonrigid CT–MR registration can provide an efficient tool for determining the geometric distortions in head and brain MRI. Our aim was to assess the geometric accuracy of our imaging center’s MRI scanners and to develop a robust QA methodology for the task. The primary purpose of the QA was to investigate, which scanners are preferable for neurosurgery planning. We also explored the suitability of low-end 3D printing as a part of the phantom manufacturing process as it could allow versatile and novel designs in the future.

## Materials and methods

**Fig. 1:**
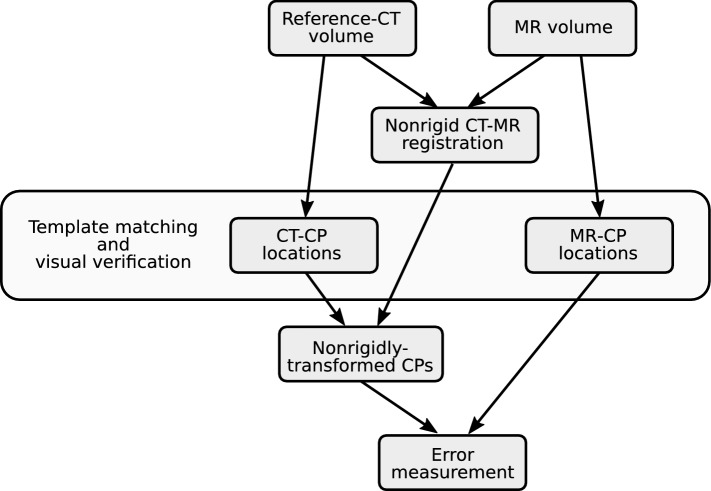
The image processing pipeline used for validating the proposed nonrigid-image-registration method. Here the MR volume is initially rigidly-registered to the CT volume and CP-CT locations are only determined once

We constructed a portable MR phantom to fit in most common head coils with a 3D-printed grid structure. We imaged the phantom with a CT and 12 MRI scanners, and reconstructed the MR image volumes both with vendors’ 3D geometric-distortion correction and without it. A semi-automated process was used to create a visually-verified reference: we localized the grid vertices in the MR and CT images with a template-matching reference-method and manually adjusted the locations when required. We then performed the nonrigid registrations of the CT and MR images with varying parameters. We compared the reference method with the values obtained via the nonrigid CT–MR registrations to find the optimized parameters for the registration and to validate the proposed manual-interaction-free procedure. The image processing steps are illustrated in Fig. 1.

### 3D phantom

**Fig. 2:**
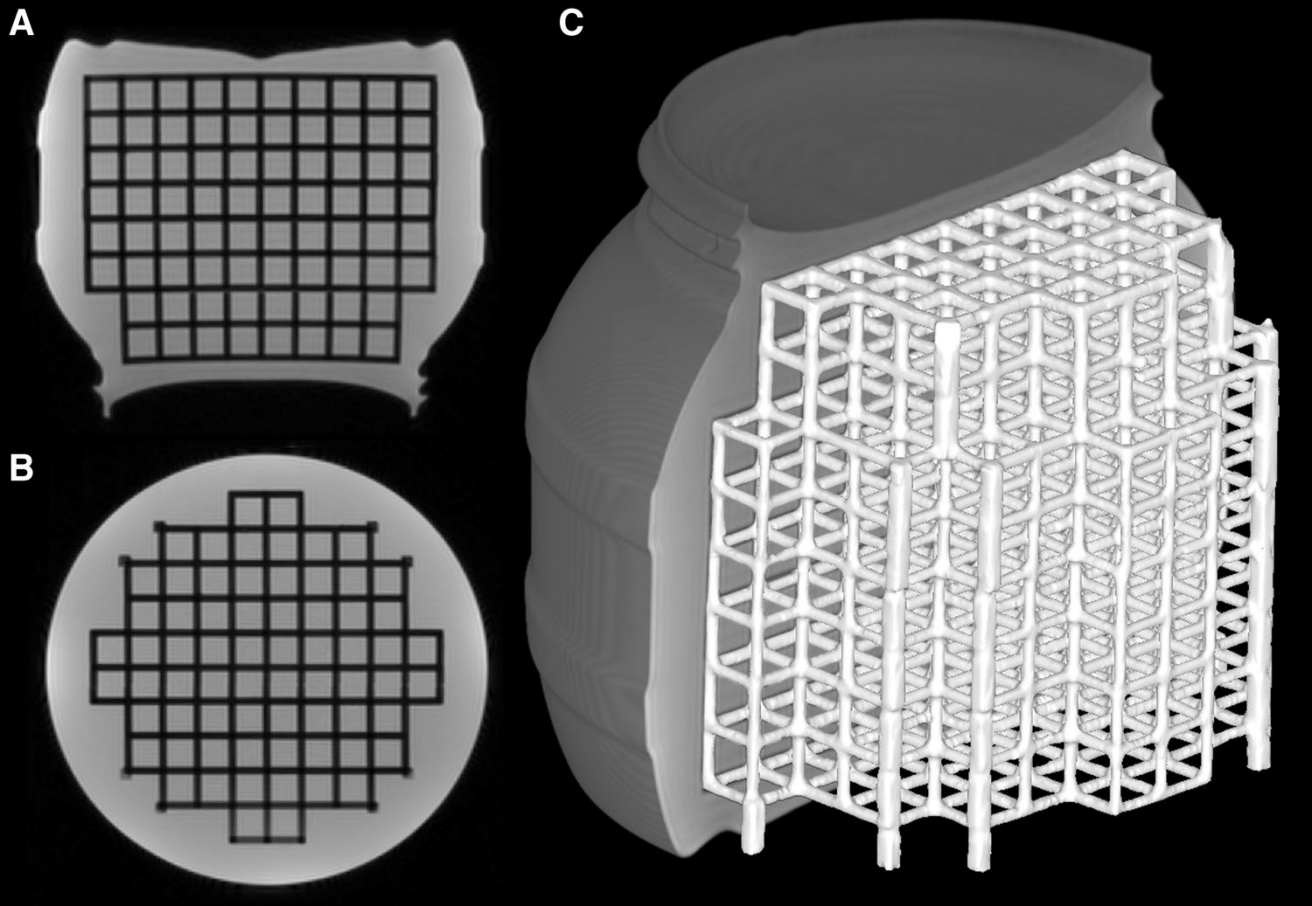
Coronal (a) and axial (b) slices, and the surface rendering (c, half of the container visible) of the MR image volume of the 3D-printed phantom structure submerged in paraffin oil

We constructed the phantom by piecing together a grid inside a wide-necked drum (CurTec International, Rijen, The Netherlands) of height 17 cm (171 mm), diameter 20 cm (198 mm), and volume 3.6 L. The drum neck opening was 14 cm (136 mm) in diameter, therefore the grid was 3D printed in 14 pieces and glued together inside the drum. We printed the grid parts from polylactide filament with two 3D printers (Ultimaker 2+ and Ultimaker 3 Extended, Ultimaker B.V., Geldermalsen, The Netherlands) to hasten the printing. The full extent of the grid was 12 cm in height and 15 cm × 15 cm in width, and the grid had 673 vertices that we treated as CPs. The solid bars forming the grid were 3 mm thick (limited by the material rigidness and the 3D-printing technique) and the vertices were 15 mm apart (similar spacing to Wang et al. [3]). We filled the drum with off-the-shelf paraffin oil. Figure 2 shows a coronal and an axial slice, and a partially surface-rendered MR image of the phantom.

### Image acquisition

We imaged the phantom with 12 clinical MRI scanners from three different vendors, of which eight were 1.5 T and four were 3 T scanners. With each scanner, we placed the phantom in a standard head coil as straight as possible using lines on the phantom and the scanner laser system. We set the scanner isocenter to the coil markings likewise to patient scans. For the image acquisition, we used sagittal T1-weighted 3D gradient-echo sequences that are in clinical use for heads and brain imaging on the corresponding scanner; Table 1 shows the scanner information and the sequence parameters. The image volumes were reconstructed with and without a vendor’s 3D geometric-distortion correction. On two scanners (A and B), the distortion correction was not a user-defined setting, and only single reconstructions were obtained for these scanners. In addition, we imaged the phantom with a CT scanner (SOMATOM Definition Edge, Siemens Medical Systems, Erlangen, Germany) using imaging parameters 120 kVp and 191 mAs, and reconstructed the image volume using pixel size 0.4 × 0.4 mm^2^, slice thickness 0.5 mm, 512 × 512 image matrix, and general-purpose soft-tissue J45s-kernel. After eight months, the phantom was re-imaged with the same CT scanner using the same protocol to assess the temporal stability of the 3D-printed structures.

**Table 1:** MRI scanner information and sequence parameters

Scanner	Year of installation	Field strength (T)	TR (ms)	TE (ms)	Flip angle (^o^)	Pixel BW (Hz/px)	Acquisition matrix	Slice thickness (mm)	Pixel size (mm^2^)	FOV (mm^2^)
A	2000	1.5	28.2	5.7	30	54	256 × 256	2.0	1.0. × 1.0	250 × 250
B	2009	1.5	9.6	3.1	20	61	256 × 256	1.0	0.5 × 0.5	256 × 256
C	2010	1.5	25	4.6	30	140	256 × 256	1.0	0.9 × 0.9	240 × 240
D	2018	1.5	7.6	3.5	8	217	256 × 230	1.0	1.0 × 1.0	256 × 256
E	2006	1.5	2200	2.5	8	170	256 × 256	1.0	1.0 × 1.0	250 × 250
F	2016	1.5	2200	2.5	8	170	256 × 256	1.0	1.0 × 1.0	250 × 250
G	2017	1.5	2200	2.5	8	170	256 × 256	1.0	1.0 × 1.0	250 × 250
H	2014	1.5	2050	2.9	15	130	256 × 232	1.0	1.0 × 1.0	232 × 256
I	2016	3	2000	2.7	10	150	256 × 256	1.0	1.0 × 1.0	256 × 256
J	2010	3	1900	2.7	9	170	256 × 246	1.2	0.5 × 0.5	250 × 250
K	2017	3	1800	2.4	8	200	320 × 280	0.8	0.8 × 0.8	222 × 254
L	2018	3	6.2	2.7	8	289	244 × 244	0.9	0.4 × 0.4	220 × 220

*TR* repetition time, *TE*  echo time, *BW*  bandwidth, the pixel size is the reconstructed image pixel size, *FOV* field-of-view

### Image preprocessing

An initial rigid registration of the MR and the reference-CT images was performed with 3D Slicer image processing platform [21] and BRAINSFit [22] module. We assumed that the effect of the geometric distortions on the phantom angular alignment were negligible near the center of the phantom and limited the registration to a spherical volume of 27 mm radius around the phantom center. Following the alignment, all the MR images were resampled to the reference-CT image’s resolution. We then confirmed the registrations visually.

### Template-matching reference-method

Our reference method was analogous to that described by Jafar et al. [6]. We localized the CPs (grid vertices) in both the MR and reference-CT images with a MATLAB code (The MathWorks, Natick, Massachusetts, USA). The template matching used a binary template of size 7.5 × 7.5 × 7.5 mm that enclosed a vertex formed by 3-mm-thick wires, and the matching was executed with normalized cross correlation (code obtained from [23]). The correlation images were thresholded, and the center-of-mass of individual elements in the resulting binary images yielded the locations of the CPs. The MR-CPs were matched with closest CT-CPs. Finally, we verified the CP locations visually in 3D Slicer and manually corrected them when needed. These CP locations are hereafter treated as reference measurements.

### Nonrigid-image-registration method

Following the initial alignment, the reference-CT image was registered to the MR images with a nonrigid-registration-toolbox elastix [24, 25]. For the registration, we used B-spline transforms [26] and a mask enclosing the grid in the CT image. We chose the constant parameters for the nonrigid registration, as well as the ranges of varying parameters, as recommended in the elastix-manual [27] and used in the previously reported parameter sets [28, 29]. The registration was fully image based; neither prior knowledge of the CPs nor manual interactions was used in this stage.

We varied the following parameters: final grid spacing (FGS), number of resolutions (NR), number of spatial samples (NSS), and maximum number of iterations (MNI). The ranges of parameter spaces were: FGS = [5 mm, 60 mm]; NR = [1, 6]; NSS = [1000, 7000]; and MNI = [100, 1000]. Firstly, we changed the FGS value, so that NR was four, NSS was 3000, and MNI was 500, as suggested in the manual. Secondly, we considered NR, thirdly NSS, and finally MNI. When a local optimum was found, we fixed this value for the rest of the registrations. Finally, we redid the FGS search with NR of three and the optimal parameters for NSS and MNI.

The transformations obtained from the nonrigid registration were applied to the CT image’s reference-CP locations. The nonrigidly transformed CP locations were compared to the visually-verified CP locations of the corresponding MR image; our error measure was the mean distance of the reference-MR-CPs and the nonrigidly transformed CT-CPs averaged over all the scanners and acquisitions. We chose the error measure minima as the optimal parameter values for the nonrigid registration.

**Fig. 3:**
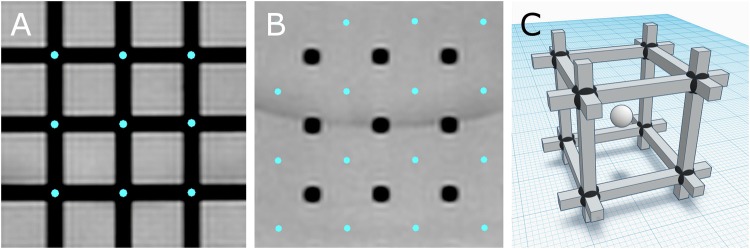
The control points (**a**) and the midpoints (**b**) in 2D planes and in a demonstrative 3D model (**c**; not-to-scale, black spheres and a white sphere, respectively)

A high number of degrees-of-freedom in the nonrigid registration may result in overfitting. This overfitting could manifest itself as unphysical or quickly alternating displacement field “ripples” in the regions of low information (i.e. the uniform background between the phantom grid wires). We assumed that the geometric distortions were smooth inside the phantom and nearly linear between neighboring grid vertices. To verify that no overfitting was happening, we recorded the midpoints of eight neighboring CPs in the reference method and compared them to the corresponding nonrigidly-transformed midpoints likewise to the actual CPs. Figure 3 shows the relation between the CPs and the midpoints; a total number of 432 midpoints per each investigated MR image were used.

### Method comparison and scanner performance

We applied the optimized nonrigid-registration method and the template-matching method to the 22 MR volumes and compared the CP displacement distributions’ maxima, means, medians, and first and third quartiles. We deemed statistic differences below half an image pixel size non-significant and regarded scanners with sub-millimeter geometric distortions as high-performing. We repeated the CT-CP localization (using both the template-matching and nonrigid-registration methods) for the second CT scan and compared the two CT volumes to affirm the stability of the grid structures over time.

## Results

### Nonrigid-registration parameters

The optimal values for the nonrigid registration with elastix-toolbox from the searched parameter space were FGS = 30 mm, NR = 3, NSS = 3000, and MNI = 500. The optimized values of NSS and MNI were the same as the initial values of these parameters. With the optimized parameter values, the mean distance between the reference-MR-CPs and the nonrigidly-transformed CT-CPs was less than (0.17 ± 0.02) mm.

**Fig. 4:**
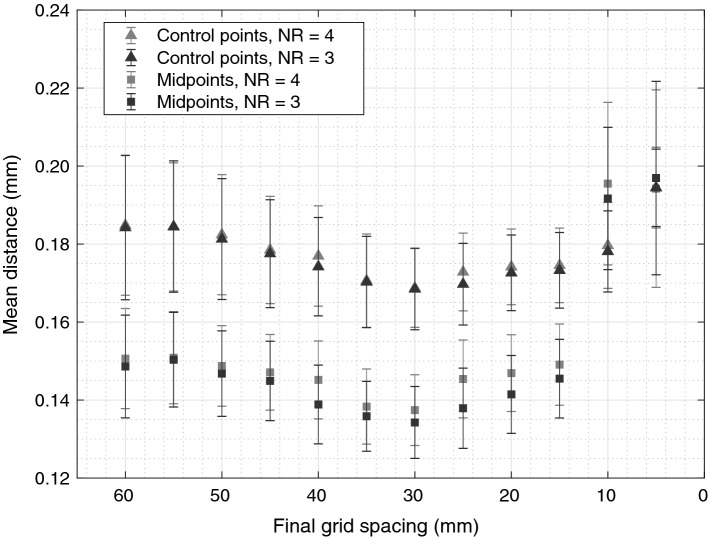
FGS was varied from 5 to 60 mm. The registration was performed on all 22 acquisitions from which the mean and the standard deviation of the distance between MR-CPs and the nonrigidly-transformed CPs was calculated. FGS of 30 mm minimizes the mean distance

Figure 4 shows the mean distance between the reference and proposed methods as a function of FGS, when NR is three and four. The FGS of 30 mm was optimal for our setup, although any of the tested values of 15 mm and higher inflicted only a miniscule absolute error. A shorter grid spacing than 15 mm resulted in overfitting seen as increased CP and midpoint errors.

**Fig. 5:**
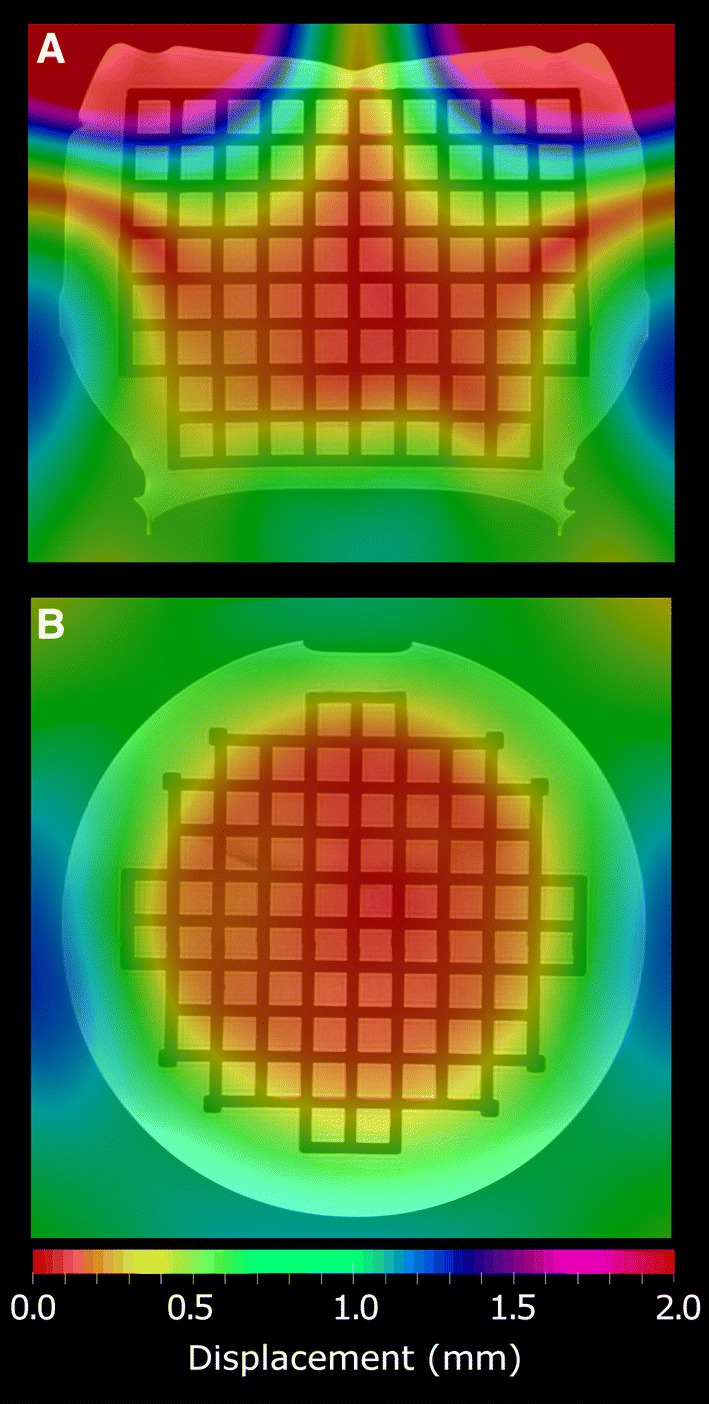
Coronal (**a**) and axial (**b**) slices of the displacement field from the nonrigid-image-registration of the non-corrected acquisition of scanner L

The changes in the mean CP distances were minimal as a function of MNI and NSS, if the parameter values were at least what was recommended in the elastix-manual. The maximum CP distance for a single acquisition changed less than 0.03 mm when NSS was above 2000 and less than 0.05 mm when MNI was above 200. When NR was on the range from two to five, the maximum distance varied within 0.05 mm, excluding scanner A, for which NR of five overestimated the maximum distance to approximately 100 mm. Moreover, NR of six yielded the maximum distance of 30–80 mm for all the acquisitions. Figure 5 shows an example of the displacement field obtained with the optimized parameters for a single acquisition; the field is extrapolated outside the grid.

### Geometric distortions and method agreement

**Fig. 6:**
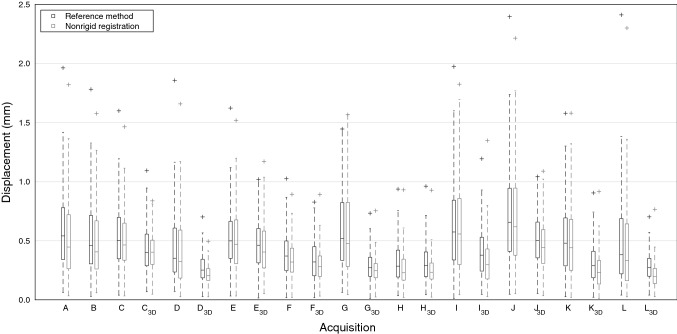
The CP displacements of each 12 scanners (A–H are 1.5T, and I–L are 3T) with non-corrected and 3D-corrected (subscript 3D) image volumes. The reported values are the reference method (template matching with manual corrections) and the proposed nonrigid-image-registration method. Here the central mark indicates the median, box defines range from the first to third quartile, and “ + ” marks a maximum outlier (i.e. maximum displacement)

Figure 6 shows the distributions of the absolute CP displacements from their true positions for the 12 scanners. The distributions were obtained with both the visually-verified template-matching reference-method and the nonrigid-image-registration method for both the non-corrected and 3D distortion-corrected volumes. The nonrigid-registrations parameters were the optimized ones (NR = 3, FGS = 30 mm, NSS = 3000, and MNI = 500). The maximal distortions using the proposed method ranged from 0.50 to 1.35 and 0.89 to 2.30 mm in corrected and non-corrected volumes, respectively. The mean distortions ranged from 0.22 to 0.46 and 0.27 to 0.72 mm, respectively.

Scanner A had the largest difference between the means of the proposed and reference methods (0.06 mm). In addition, scanner A had the largest difference in the medians and the first quartiles (0.09 mm and 0.08 mm, respectively), whereas the 3D-corrected acquisition of scanner I had the largest difference in the third quartiles (0.10 mm). Three scanners had larger difference than 0.07 mm in the medians, 0.06 mm in the first quartiles, and 0.08 mm in the third quartiles. The difference in the means, the medians, the first quartiles, and the third quartiles was less than 0.05 mm in twelve, fifteen, fifteen, and ten acquisitions, respectively. The 3D-corrected acquisition of scanner C had the largest difference in maximum displacements between the two methods (0.26 mm). Other acquisitions had a difference of 0.21 mm or less in the maximum displacements; eight acquisitions have this difference less than 0.10 mm. All maximum displacement locations were on the edges of the phantom grid. All the discrepancies were below half the pixel value and therefore considered non-significant.

In the distributions obtained with the reference method, the maximum displacements decreased on average by 0.77 mm when the 3D correction was applied, and the maximum decrease was 1.71 mm. Consistently, the mean displacements decreased on average by 0.17 mm, and the maximum decrease was 0.30 mm. Apart from the scanner H, all non-corrected acquisitions, as well as acquisitions with scanners A and B, had a maximum displacement above 1.0 mm. After the 3D distortion correction, the maximum displacement of five scanners out of nine was reduced to below 1.0 mm as four stayed above 1.0 mm. The maximum displacement of scanner H with or without correction remained below 1.0 mm.

In the distributions obtained with the nonrigid-registration method, the maximum displacements decreased on average by 0.68 mm (maximum 1.54 mm) with the 3D distortion correction; the mean displacements decreased on average by 0.18 mm (maximum 0.33 mm). Differing from the reference method, the maximum displacement of the non-corrected acquisition F was below 1.0 mm, and the maximum displacement of scanner C was reduced below 1.0 mm in the 3D-corrected acquisition.

The CT scan of the constructed phantom was repeated after eight months to monitor the temporal stability of the 3D-printed grid structures. Between the two CT acquisitions the mean (and maximum) displacement of CP locations was 0.20 mm (0.30 mm) using the template-matching method and 0.15 mm (0.20 mm) using the nonrigid-registration method. Upon visual inspection of the CT images, no additional defects or deformations was observed.

## Discussion

In this study, we aimed to develop an easy-to-perform QA protocol for measuring MRI scanner’s geometric accuracy and for evaluating the suitability of the MRI scanner performance in the neurosurgery planning. The nonrigid image registration allows the measurement of the geometric distortions in the MR images of a simple phantom, when a geometrically-accurate reference-CT is available. The proposed method can be applied to QA purposes; most of the MR acquisitions in our study had maximum distortions of sub-millimeter scale, but some acquisition yielded significant distortions.

Our results show, that the CP displacement distributions of the template-matching reference-method and the nonrigid-image-registration method with the optimized registration parameters are very similar for each acquisition (Fig. 6). The most important registration parameter to optimize is FGS when elastix-toolbox is used, as too short FGS causes overfitting in the registration. Otherwise, the elastix-manual offers well suited parameters; our initial values of NSS and MNI proved to be the optimal ones, too.

According to ACR’s MRI accreditation program [20], the maximum allowed deviation from a known phantom structure length is ± 2.0 mm, or approximately ± 1%, whereas the American Association of Physicists in Medicine acceptance criteria is ± 2% (and preferably less for treatment planning) [30]. Two of the 3T scanners had the maximum CP displacement over 2.0 mm without the 3D correction, but the correction reduced the maximum value to 1.0 mm or below. Overall, the maximum displacement was less than 2.0 mm in all the rest of the acquisitions, and less than 1.0 mm in the 3D-corrected acquisitions of seven scanners. The 3D correction might not be on by default in vendor-provided sequences and should be considered when images are used for the treatment planning. Some vendors allow the correction to be applied retrospectively.

The CP localization method must be precise to achieve accurate geometric distortion measurements, which must be considered in the phantom design and the image-processing pipeline. For example, spherical markers are harder to detect as accurately as grid vertices because the markers need to be large to produce enough MR contrast, according to Wang et al. [3]. A manual CP localization would be time consuming and could introduce user-related errors, whereas our template-matching code required a fair amount of manual adjustments both in designing the template and in correcting the proposed CP positions. The usage of the full- or partial-image-based nonrigid registration eliminates the need for individual CP localization in the image-processing pipeline. The nonrigid registration requires identifiable features in the images, but the method is less dependent on the phantom design than many previous studies on geometric accuracy.

**Fig. 7:**
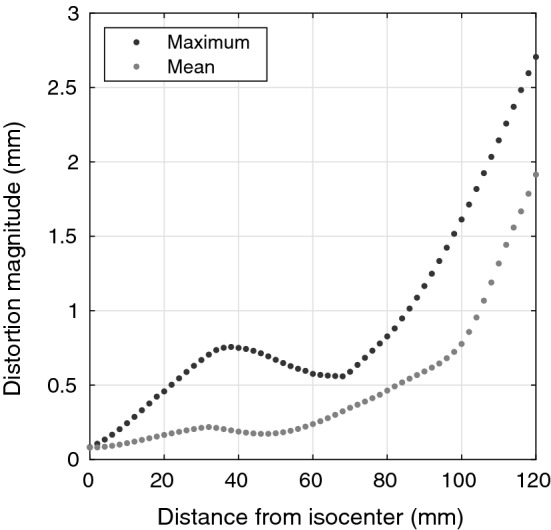
A plot of distortion magnitude as a function of distance from the phantom isocenter, which is suitable for an easy-to-interpret QA report

The proposed phantom can be used in an inter-scanner comparison, in setting objectives and repeatable performance requirements, and in a scanner’s QA over extended time assuming the phantom does not change in time. Periodical CT acquisitions can be used in phantom QA with the same nonrigid methodology as described above but for a CT–CT registration. As Table 1 shows, the proposed methodology and the obtained parameters were suitable for a range of scanner generations and manufactures and, to a limited extent, sequence types. We did not investigate other sequence types or contrasts, but the method could be adapted to other sequences, such as T2-weighted or fluid-attenuated inversion recovery sequences. The exact positioning of the phantom in the scanner is not crucial if the MR system coordinates are maintained: the QA methodology is based on the distribution of the absolute distortion magnitudes. An example of presenting the results of a single QA measurement, where the displacement field is reduced to a one-dimensional graph, is shown in Fig. 7. A benefit of the precise positioning is the improved reproducibly and comparability of the results.

Portability and mimicking the exact clinical scan setup (i.e. scan in a head coil) were the primary drivers of the phantom design. Even though our in-house phantom is adequate to distinguish the best and the worst performing scanners, a more complex phantom could be constructed in the future. A better fit into the head coil and a greater grid coverage would be desirable and could be achieved with an anatomically-shaped phantom. Narrower rods and denser vertices could improve the accuracy and the statistical power especially at the regions where distortions are the greatest. In a surgery planning station, the CT–MR registration can be highly dependent on the bony structures, which underlines the importance of better investigative coverage.

The 3D printing is a versatile manufacturing and prototyping method. The phantom and the 3D-printed structures can easily be modified (re-printed) to meet new and novel requirements. The limited accuracy of a consumer-level 3D printer can be tolerated using the CT as the ground truth. The 3D-printed polylactide structure may degrade over time, and the phantom’s geometric accuracy may need to be re-verified periodically if longitudinal monitoring is to be carried out. The filling material (paraffin oil in our case) may also affect the deterioration rate. Over the period of eight months, the maximum observed CP displacements between the two CT scans were well under the CT voxel-size of 0.4 × 0.4 × 0.5 mm, and the phantom can be deemed reasonably stable at this time scale. The discrepancy between the result from the nonrigid-registration and the template-matching methods, as seen in the MRI distortion measurements, was of the same order with the CT volumes’ CP displacements. A phantom constructed with high-precision commercially-manufactured structures and known geometry would remove the requirement for the reference-CT. In this case, the volumetric nonrigid-registration method could be applied to a virtual phantom.

The MRI head and brain scans are acquired in the center of a scanner’s bore where the geometric distortions are the smallest. Together with the advanced shimming of the modern scanners, the distortions in the head images are mostly caused by tissue-related factors. Compared to the proposed phantom, an anthropomorphic phantom with a possibility of precise and repeatable positioning could allow more realistic evaluation of the susceptibility effects and, consequently, the unwarping of patient images. The displacement field obtained from a QA measurement could be used with a CT–MR registration of the patient images to assess the magnitude of the patient-specific distortions.

Even without an improved phantom, the nonrigid CT–MR registration could be tested in unwarping of patient images, if a CT scan of the patient is available. Similarly, an MR volume could serve as the reference volume, if a patient is imaged with two different MRI scanners; the correction could be applied to the volume with greater distortions. In addition to anatomical scans, the nonrigid-registration method and the proposed phantom could roughly unwarp super-fast acquisitions with greater distortions, for example echo planar imaging acquisitions.

The nonrigid-image-registration-based QA-methodology provides an analysis tool for measuring geometric accuracy in MRI comparable to manually locating the CPs. The proposed method does not set explicit requirements for the test object apart from the desired target structure spacing, coverage and suitable contrast. The presented procedure is easy to perform in a clinical setting once the necessary software and the registration parameters are established. In addition, only few instructions are needed; the QA procedure could easily be fully automated.
